# Haploidentical Allogeneic Stem Cell Transplantation as a Superior Alternative for Patients With Mismatch Donors—A Single Center Experience in 152 Patients

**DOI:** 10.1002/jha2.70012

**Published:** 2025-03-04

**Authors:** Paul Jäger, Benno Biermann, Nora Liebers, Felicitas Schulz, Ben‐Niklas Baermann, Sören Twarock, Stefanie Geyh, Kathrin Nachtkamp, Patrick Tressin, Annika Kasprzak, Felix Matkey, Titus Watrin, Malika El Yaouti, Ulrich Germing, Sascha Dietrich, Guido Kobbe

**Affiliations:** ^1^ Department of Hematology Oncology and Clinical Immunology Medical Faculty and University Hospital Düsseldorf, Heinrich Heine University Düsseldorf Duesseldorf Germany; ^2^ Medical Faculty and University Hospital Düsseldorf, Institute of Translational Pharmacology Heinrich Heine University Düsseldorf Duesseldorf Germany

**Keywords:** BMT, cellular therapies, hematological malignancies, transplant

## Abstract

**Background:**

Allogeneic hematopoietic stem cell transplantation (allo‐SCT) is a key treatment for hematologic malignancies, but donor selection impacts outcomes.

**Results:**

In a cohort of 152 patients undergoing allo‐SCT from 2012 to 2023, haploidentical donors with post‐transplant cyclophosphamide (PTCy) showed superior survival compared to 9/10 mismatched unrelated donors (MMUD). Cox regression analysis revealed that patients not in complete remission (CR) before transplantation particularly benefited from haplo donors, while those with 9/10 MMUD and lacking CR had worse outcomes.

**Conclusion:**

These results highlight the importance of donor selection, suggesting that haplo donors with PTCy may be preferable for patients not in CR, necessitating alternative approaches for others.

**Clinical trial registration:**

The authors have confirmed clinical trial registration is not needed for this submission.

## 1 Introduction

Allogeneic hematopoietic stem cell transplantation (allo‐SCT) is an important treatment option for high‐risk hematologic malignancies, but success hinges on finding HLA‐compatible donors. When fully matched donors are scarce, options like partially HLA‐matched related (haploidentical) and mismatched unrelated donors (9/10 MMUD) expand transplant accessibility. However, challenges such as graft‐versus‐host disease (GvHD) and relapse persist with mismatched donor allo‐SCT, necessitating research into effective prevention methods [1, 2].

Recent advances, notably post‐transplant cyclophosphamide (PTCy), have renewed interest in haploidentical donors, improving outcomes by overcoming previous compatibility issues [1]. In contrast, 9/10 MMUD patients typically undergo T‐cell depletion with antithymocyte globulin (ATG), with PTCy showing promise for future use [3, 4].

When both haploidentical and 9/10 MMUD donors are available, selecting the best donor requires considering individual patient factors and treatment needs. While haploidentical donors may offer advantages in certain scenarios, specific indications for preference need clarification [5, 6, 7].

Based on our single‐center experience, we offer valuable insights into the donor selection process for patients, taking into account factors such as remission status and other relevant considerations like age, EBMT Gratwohl score, performance of total body irradiation (TBI) and disease entity. This contribution enhances the decision‐making process and aids in optimizing donor choices for improved patient outcomes.

## 2 Methods

**FIGURE 1:**
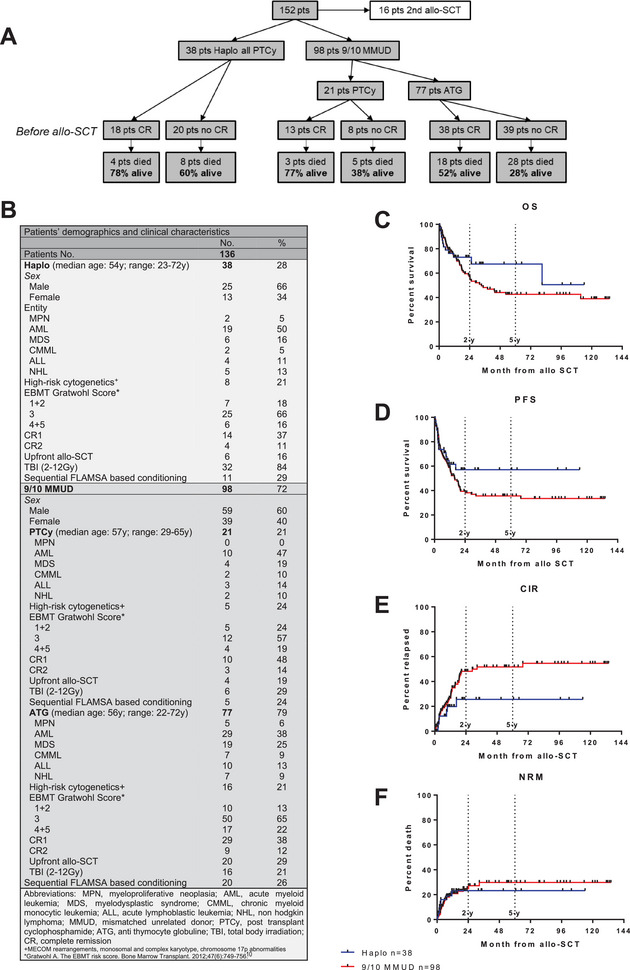
(A) CONSORT diagram. (B) Patient´s demographics and clinical characteristics. Kaplan–Meier survival curve with overall survival (OS) (C) and progression free survival (PFS) (D) month from allo‐SCT of patients with 9/10 MMUD versus patients with haplo donors. Cumulative incidence of relapse (CIR) (E) and non‐relapse mortality (NRM) (F) as competing risks month from allo‐SCT of patients with 9/10 MMUD versus patients with haplo donors.

We retrospectively analyzed 152 patients with various hematological malignancies who underwent allo‐SCT from mismatched donors at our center between 2012 and 2023 (haplo PTCy (50 mg/kg d+3 and d+4) *n* = 38, 9/10 MMUD ATG (rabbit ATG 20 mg/kg per day, administered over 3 consecutive days (total dose: 60 mg/kg)) *n* = 77, 9/10 MMUD PTCy (50 mg/kg d+3 and d+4) *n* = 21). All individuals received identical diagnostic work up and supportive care independent from donor source or GvHD prophylaxis. Patients with 2nd or 3rd allo‐SCT were excluded from further analysis (Figure 1A). Disease entities, disease and transplant characteristics, EBMT scores, and demographic characteristics were evenly distributed among the groups (Figure 1B). Complete remission at the time of transplant was defined as follows: in AML, MDS, and CMML (the latter also requiring monocytes <1 × 10⁹/L), CR was characterized by blasts <5% and normalized blood counts. In MPN, CR was defined by normalized blood counts, spleen regression, and the absence of molecular markers. In ALL, CR was achieved when blasts <5%, normalized blood counts, and MRD‐negativity were confirmed. For NHL, CR was defined by the absence of FDG activity on PET‐CT and/or no lymph nodes >1.5 cm with the resolution of all lesions on CT, along with normalized blood counts and no remaining lesions. Overall survival (OS) and progression‐free survival (PFS) were calculated from allo‐SCT to death from any cause or last follow‐up, and until progression to molecular or hematologic relapse or death, respectively. Cumulative incidence of relapse (CIR) and non‐relapse mortality (NRM) were calculated as competing risks. Statistical analyses were conducted using GraphPad Prism® 5.01 and SPSS Statistics for Windows, with significance set at *p* < 0.05. Further details are provided in the figure legend.

The study was approved by our local ethics committee (study number 2023–2474)

## 3 Results

After a median follow‐up of 63 months (range: 6–120 months) post allo‐SCT, patients who underwent haploidentical donor transplantation demonstrated better two‐year overall survival (2y‐OS) and two‐year progression free survival (2y‐PFS) compared to those with 9/10 MMUD with 73% versus 56% (Figure 1C) and 57% versuss 39% (Figure 1D), respectively. Additionally, the CIR was observed to be lower in the cohort of patients with haploidentical donors compared to those transplanted with 9/10 MMUD with a 2y‐CIR of 26% versus 48% (Figure 1E), whereas the NRM was similar with a 2y‐NRM of 23% versus 27% (Figure 1F).

**FIGURE 2:**
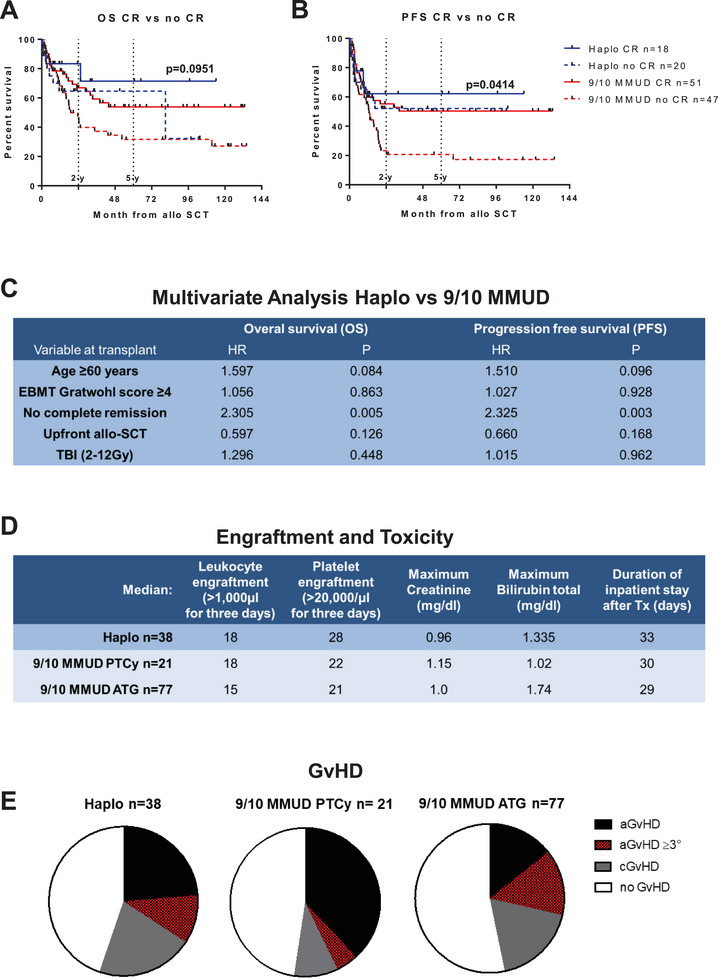
Kaplan–Meier survival curve with (A) overall survival (OS) and (B) progression free survival (PFS) month from allo‐SCT of patients with 9/10 MMUD versus patients with haplo donors, split by remission status before transplant. (C) Multivariate Cox regression analysis affecting overall survival (OS) and progression free survival (PFS) of patients with haplo donors versus patients with 9/10 MMUD. Hazard ratio and *p*‐values are given. (D) Table with engraftment and toxicity data. (E) Pie charts demonstrating the occurrence of GvHD in the respective group.

Haploidentical donor recipients transplanted in complete remission (CR) exhibited a 2y‐OS and a 2y‐PFS of 83% and 62%, respectively. Interestingly, patients who received transplants from 9/10 MMUD while in CR had comparable 2y‐OS and 2y‐PFS rates of 67% and 55% to patients with haploidentical donors who had active disease at the time of transplantation (2y‐OS 65% and 2y‐PFS 52%). Conversely, individuals who underwent transplants from 9/10 MMUD with active disease experienced significantly lower 2y‐OS and 2y‐PFS rates of 47% and 23%, respectively (*p* = 0.0414) (Figure 2A,B). Multivariate Cox regression analysis showed that remission status prior to allo‐SCT had the greatest impact on OS and PFS, with significant advantages for superior OS and PFS for patients who received a graft from a haploidentical donor (HR OS: 2.305, *p* = 0.005; HR PFS: 2.325, *p* = 0.003, Figure 2C). Factors such as age ≥60 years, upfront transplantation, a high EBMT score or the performance of TBI had no significant influence on OS and PFS (Figure 2C).

As anticipated, distinct disease entities exhibited varied outcomes, yet they were evenly distributed across the main groups (Figures 1B and S1A). Given the relatively small sample size and our primary focus on remission status and other variables, patients were analyzed collectively.

PTCy appears to be a useful alternative in patients with 9/10 MMUD and offers potential prognostic advantages in terms of 2y‐OS (71% vs. 54%) compared to patients with 9/10 MMUD receiving ATG (Figure S1B), although no statistical significance was reached. Organ toxicities were similar between the three groups; however, white blood cell engraftment (>1000/µL) was delayed by a median of 3 days and platelet engraftment (> 20000/µL) by a median of 6 days in the haplo group. As a result, the median hospital stay was 3 days longer in the haplo group (Figure 2D). In transplants from 9/10 MMUD, the administration of PTCy did not result in an overall reduction in GvHD activity but a decrease of the incidence of chronic and severe acute GvHD (Figure 2E).

## 4 Discussion

Analyzing 152 patients who underwent transplants from mismatched donors at our center over the past decade, we provide crucial insights into outcomes, informing clinical decisions and optimizing patient care. Notably, haploidentical donor transplantation demonstrated superior 2y‐OS and 2y‐PFS compared to 9/10 MMUD, aligning with previous studies advocating for haploidentical transplantation with PTCy for GvHD prophylaxis [6, 7, 8].

Our findings underscore the impact of pre‐allo‐SCT remission status on survival outcomes, particularly favoring haplo donors for patients not in remission. Patients transplanted in CR exhibited significantly better OS and PFS rates, consistent with literature emphasizing the importance of disease status in allo‐SCT outcomes [7, 9]. Haploidentical transplantation appears to offer better disease control, especially in patients not in CR.

Furthermore, our results confirm the benefit of PTCy in 9/10 MMUD and are consistent with previous studies involving larger cohorts [4]. This prompts the question of whether potential negative effects of active disease status pre‐transplant with 9/10 MMUD can be mitigated by the use of PTCy. Our experience indicates that both haploidentical and 9/10 transplants are equally feasible, the latter with either ATG or PTCy, with no significant difference in NRM. Notably, there were no cases of graft failure in the haplo PTCy group, which may be attributed to our practice of incorporating at least 2 Gy TBI into the conditioning regimen. When comparing ATG and PTCy, no disadvantages were observed with PTCy, even in older patients for whom cyclophosphamide might pose higher risks. Ongoing studies are exploring dose reductions of cyclophosphamide to further optimize patient outcomes.

It is important to acknowledge that our single‐center, retrospective study with a relatively small cohort presents potential biases, including heterogeneity in disease types, graft‐versus‐host disease (GVHD) prophylaxis, and conditioning regimens, which should be addressed in future studies involving larger, more homogeneous cohorts. In conclusion, our study suggests the potential superiority of allo‐SCT with a haploidentical donor over 9/10 MMUD, particularly for patients who are not in remission before transplantation. The impact of PTCy on survival rates in patients with 9/10 MMUD who are not in remission warrants further investigation through randomized prospective studies with well‐defined cohorts. This is crucial because outcomes are likely influenced by multiple factors, including the specific disease entity and pre‐transplant disease status. Ultimately, these findings provide important insights that can help refine donor selection strategies and improve patient outcomes in clinical practice.

## Author Contributions

P.J., B.B., and G.K. designed the research study. P.J., B.B., and G.K. performed the research. P.J., B.B., N.L., S.D., and G.K. contributed essential data. P.J., B.B., N.L., S.D., and G.K. analyzed the data. P.J., B.B., and G.K. wrote the paper. All authors critically revised the manuscript.

## Ethics Statement

The study was approved by our local ethics committee (study number 2023–2474).

## Patient Consent Statement

All patients provided informed consent for registry studies as part of their initial diagnosis process.

## Conflicts of Interest

The authors declare no conflicts of interest.

## Supporting information




**Figure S1: (A)** Kaplan–Meier survival curve with overall survival (OS) month from allo‐SCT of patients with 9/10 MMUD versus patients with haplo donors split by different disease entities. (**B**) Kaplan–Meier survival curve with overall survival (OS) month from allo‐SCT of patients with 9/10 MMUD split by application of PTCy versus ATG.

## Data Availability

Publication‐related data are available from the corresponding author on reasonable request.
