# Supplementary figures and images for: Haploidentical Allogeneic Stem Cell Transplantation as a Superior Alternative for Patients With Mismatch Donors—A Single Center Experience in 152 Patients
Source: EJHaem. 2025 Mar 4;6(2):e70012. doi: 10.1002/jha2.70012 (PMC11876775; doi:10.1002/jha2.70012)

## Slide 1
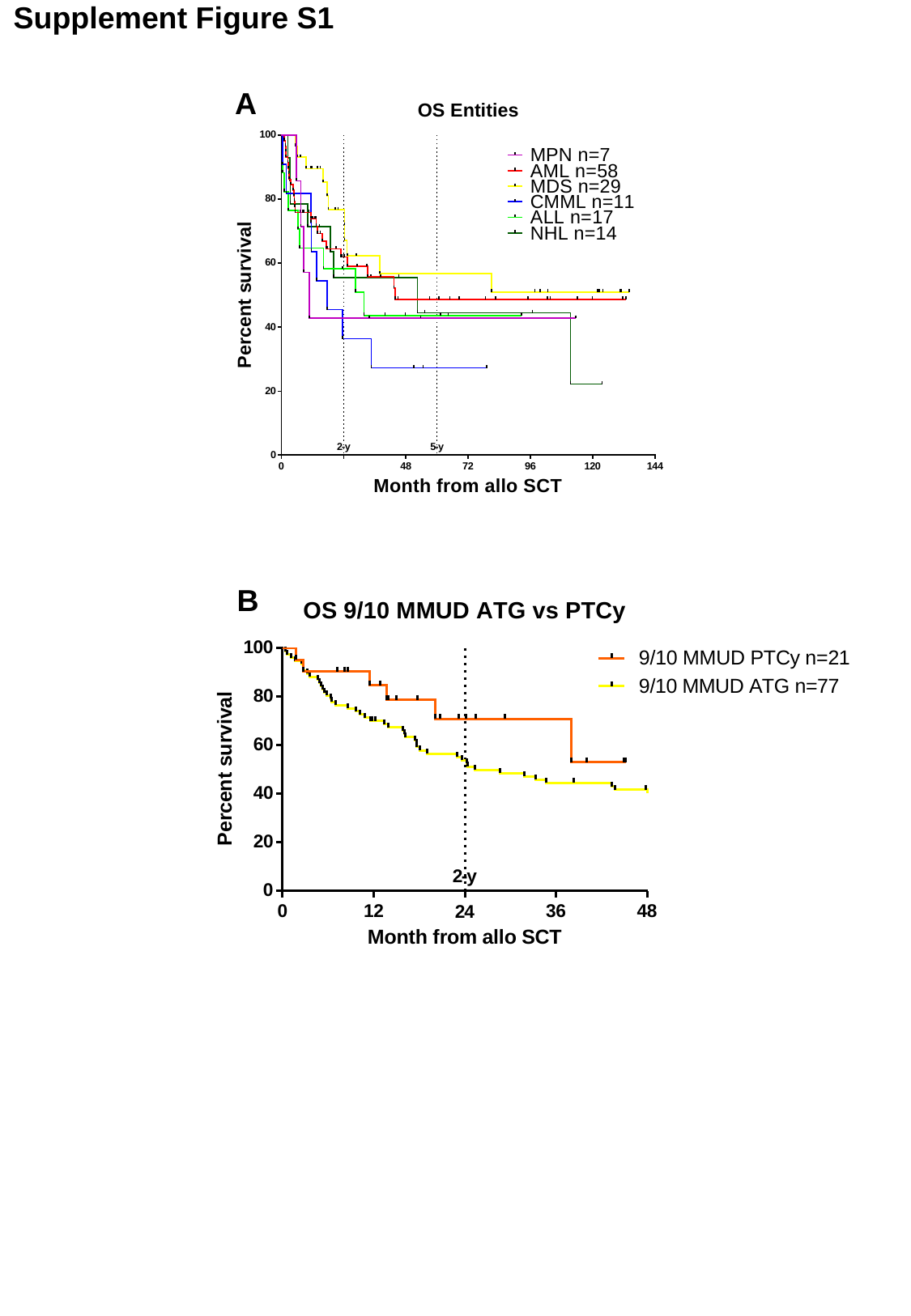

Supplement Figure S1
A
B

Supplement: Supplementary file 1 — Figure S1: (A) Kaplan–Meier survival curve with overall survival (OS) month from allo‐SCT of patients with 9/10 MMUD versus patients with haplo donors split by different disease entities. (B) Kaplan–Meier survival curve with overall survival (OS) month from allo‐SCT of patients with 9/10 MMUD split by application of PTCy versus ATG. [file JHA2-6-e70012-s001.pptx]
